# Translational Control in Liver Disease

**DOI:** 10.3389/fphys.2021.795298

**Published:** 2021-11-29

**Authors:** Alexandra Balvey, Mercedes Fernandez

**Affiliations:** Laboratory of Translational Control in Liver Disease and Cancer, IDIBAPS Biomedical Research Institute, University of Barcelona, Barcelona, Spain

**Keywords:** translation, RNA-binding proteins, CPEB proteins, liver disease, portal hypertension

## Abstract

Chronic liver disease is one of the biggest threats to public health worldwide. Worryingly, the incidence of liver disease is dramatically rising due to the aging of the population and the global epidemics of obesity. Both are major risk factors for chronic liver disease and adverse prognostic factors, causing an increase in mortality rate. It is of great concern that 80–95% of obese people have non-alcoholic fatty liver disease, the major precursor for liver failure and a global health challenge. Currently, the only curative treatment for advanced chronic liver disease is liver transplantation, which is, however, hampered by high treatment costs and the scarcity of donor organs. New strategies are therefore urgently needed to prevent and reverse chronic liver disease. And for that it is essential to understand better the molecular mechanisms underlying human disease. This review focuses on the abnormalities in the regulation of translation by RNA-binding proteins during chronic liver disease and their pathological impact on portal hypertension, fibrosis, steatosis, neovascularization, and cancer development.

## Introduction

Chronic liver diseases, along with hepatocellular carcinoma (HCC), have increasingly become a global significant health burden, affecting as much as 5.5 million people worldwide (Younossi et al., 2016; Pimpin et al., 2018). This set of diseases are characterized by decreased hepatic function as a result of chronic inflammation and repeated insults to the liver, often leading to an irreversible and fatal outcome. It can no longer be ignored that the dramatically increasing global epidemic of obesity greatly helps at fueling metabolic conditions, which will often manifest through the liver in the form of non-alcoholic or metabolic associated fatty liver disease (NA/MAFLD), predisposing to a spectrum of diseases including non-alcoholic steatohepatitis (NASH), fibrosis (Mejias et al., 2020a), cirrhosis and HCC (Younes and Bugianesi, 2018; Zhang, 2018). Under such circumstances, the liver and its cellular population are forced to undergo metabolic reprogramming to compensate the new condition. Underlying inflammation and other metabolic changes, regulation of mRNA translation through the control of poly(A) elongation has been observed as a key factor (Curinha et al., 2014; Chen et al., 2016), where the main actors have been found to be the cytoplasmic polyadenylation element binding (CPEB) family of proteins, mostly implicated in cell proliferation, tumorigenesis, invasiveness, angiogenesis and fibrogenesis. This review aims to stand out the role of key CPEB proteins in the regulation of mRNA translation under metabolic stress in the liver, contributing to gather and bring further the limited knowledge we have on the underlying molecular mechanisms, in order to find alternative approaches to treat these diseases.

## Posttranscriptional Control of Gene Expression

Gene expression regulation is an intricate, interconnected and multi-layered process involving three main players (DNA, RNA and proteins), in which every step is tightly monitored and controlled to ensure optimal cellular adaptation to the environmental and physiological demands, while remaining robust to transient perturbations (Moore, 2005). Whereas the nucleotide sequence of a gene determines the sequence of its mRNA product, and whereas an mRNA’s sequence determines the amino acid sequence of the resulting polypeptide, there is no trivial relationship between the concentration of a transcript and the concentration(s) of the protein(s) derived from it (Liu et al., 2016). Although most of the research from the last decades has focused on the first step of the pathway (the regulation of transcription), novel systematic studies quantifying transcripts and proteins at genomic scales exposed the importance of multiple processes beyond transcript concentration, contributing to establishing the expression level of a protein (McManus et al., 2015). Translational efficiency thus becomes the single best predictor of protein expression (Schwanhäusser et al., 2011), underlining the importance of the last step in the gene expression cascade.

mRNA translation is a cyclical process that has been described and investigated for many years now, identifying three steps from which initiation has been the most studied, followed by elongation and termination. Translation initiation is the most complex step and rate-limiting, involving a myriad of proteins (with new ones being linked to it as research progresses), typically divided in a standard cap-dependent initiation and an alternative cap-independent initiation. Both mechanisms have the purpose of recruiting the mRNA and assembling the ribosomal subunits so that translation can begin.

Cap-dependent translation is the most general mechanism of translation initiation (Ruggero and Shimamura, 2014). A set of proteins called eukaryotic initiation factors (eIFs) are required for the recruitment of the 40S subunit on the 5′UTR, right where the m^7^Gppp group is. Once assembled, the 40S subunit scans the mRNA until reaching the start codon (AUG), which is then pinpointed and paired with the anticodon tRNA at the peptidyl site (or P-site), culminating with the recruitment of the 60S ribosomal subunit and thus forming the whole ribosomal complex, ready to proceed to the elongation phase. However, the alternative mechanism of translation initiation, called Internal Ribosome Entry Site (IRES) mediated translation, will allow the translation of mRNAs in a cap-independent manner, sparing the need of 5′UTR recognition as well as the mRNA scanning process, by directly recruiting the 40S subunit nearby the codon where the translation must be initiated.

Translational elongation is a mechanism conserved in all kingdoms of life, assisted by a minimal set of factors (Dever et al., 2018). During this step, the ribosome reads the mRNA sequence and consequently adds the corresponding amino acids matching each codon, mediated by elongation factors eEF1A-B, eEF2, and eIF5A, which keep the cognate aminoacyl-tRNAs flowing through the A- (acceptor), P- (peptidyl) and E- (exit) sites, as the lecture proceeds.

The last step in translation is known as termination, and it is triggered when the ribosome reaches the stop codon. Two release factors (eRF1 and eRF3), with the help of GTP, constitute a ternary complex that execute the release of the nascent peptide (Hellen, 2018). The complex follows disassembly, allowing its constituents to integrate further rounds of translation in a posterior recycling phase.

Once the mRNA transcript has completed its function, it is required to undergo physiological exonuclease-mediated degradation by diverse decay pathways (Halbeisen et al., 2008), each one being carefully controlled to recognize its target mRNAs. Oddly, the major cytoplasmic mRNA degradation pathway in eukaryotes begins with shortening of the poly(A) tail by a variety of deadenylases, before the removal of the 5′cap structure by decapping enzymes Dcp1 and Dcp2 (Parker and Song, 2004). After that, the decapped intermediates are consequently digested either by Xrn1p exonuclease (5′ → 3′) or by the exosome complex (3′ → 5′).

The poly(A) tail is a dynamic structure constituted by a sequence of 200–250 adenine residues (its length varying among species), found at the 3′UTR end of all nuclear transcribed eukaryotic mRNAs. One of its main essential functions is blocking mRNA degradation by ubiquitous exonucleases, and thus providing an additional regulatory opportunity to extend (or shorten) the transcript’s life while remaining in the cytoplasm by just allowing the addition or deletion of adenine residues from the 3′end, respectively. Moreover, a long poly(A) tail will allow stabilization of the mRNA during translation by circularization, assisted by initiation factors eIF4E and eIF4G, and PABP, all of which are target of a number of factors that will stimulate or inhibit the translation of specific mRNAs (Fernández-Miranda and Méndez, 2012).

## RNA-Binding Proteins

The eIF4E, eIF4G, and PABP belong to the great family of RNA-binding proteins (RBPs), together with a myriad of other proteins. RBPs are central components in RNA metabolism, since they regulate all aspects in the life of mRNAs; from their synthesis, processing and maturation, to their export, stability, transport and translation, in addition to generating connections and regulatory networks between these processes, so that perturbations in the pathway can be quickly neutralized or, at least modulated. This crucial role is also played by the CPEB (cytoplasmic-polyadenylation element binding protein) family, which belongs to the RBP category as well. The first clues on the existence of this family came around the 1990s when Richter and colleagues were observing the involvement of p34^cdc2^ kinase in cyclin-mediated polyadenylation (Paris et al., 1991; Hake and Richter, 1994). They found that p34^cdc2^ kinase was phosphorylating another kinase, probably Aurora-A kinase, which in turn phosphorylated a CPE-binding protein, happening to be CPEB1, years of research after. Fast forward, up-to-date genome-wide studies found that around 20% of vertebrate genome transcripts contain CPE elements, thus turning into potential CPEB targets (Pavlopoulos et al., 2011), although the specific roles and regulation of each CPEB are still poorly understood in adult tissues.

**FIGURE 1:**
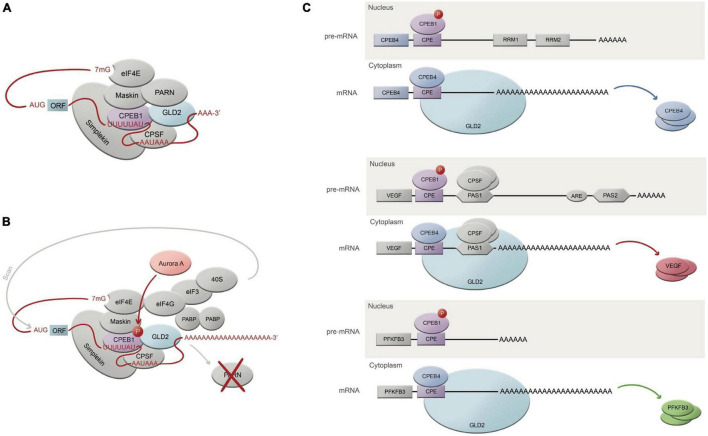
CPEB-regulated mechanism of translational activation through polyadenylation. **(A)** Translationally repressed mRNA where CPEB1 is bound to the CPE domain (U-rich UUUUUAU sequence), while at the same time, CPSF is associated to a nuclear pre-mRNA site AAUAAA. CPEB1 is at the same time interacting with GLD2 (adenylating enzyme), which is inhibited by its binding to PARN (deadenylating enzyme). PARN shortens the poly(A) tail in this translational repression state. CPEB1 is also bound to Maskin, which interacts with eIF4E, which in turn also cooperates in translational repression by taking the place of eIF4G. Simplekin acts as a platform for the protein complex tethering the repressed mRNA. **(B)** Upon Aurora A phosphorylation, CPEB1 is thus phosphorylated and activated, which will cause PARN ejection, liberating GLD2 to finally be able to resume polyadenylation. The resulting long poly(A) tail will facilitate the 40S ribosomal subunit to find it, guided by eIF4G-eIF3 complex, finally allowing translation. **(C)** Roles and mechanism of CPEB1 and CPEB4 in 3 different mRNAs related with the pathophysiology of chronic liver disease; active CPEB1 will bind to the CPE sequence in pre-mRNAs within the nucleus, allowing CPSF to cleave the hexanucleotide domain, originating the mature mRNA. Once the mature mRNA has exited the nucleus, CPEB4 will bind the CPE sequence to enhance polyadenylation and thus prioritizing the translation of poly(A)-rich mRNAs.

Cytoplasmic polyadenylation element binding are of special interest for their active role in cytoplasmic polyadenylation modulation, which they execute through binding specific structural elements on targeted mRNAs and by interacting with other proteins (Figures 1A,B). Intriguingly, CPEBs have the ability to both activate and repress translation by turning on polyadenylation and deadenylation, respectively; they do so by sticking to specific sequences located on the 3′UTR end of the transcripts, the most common one being the CPE (cytoplasmic polyadenylation element), an AU-rich domain (Belloc and Méndez, 2008). Whether they bind with higher or lower affinity to these sequences, and whether they end up activating or repressing translation, will greatly depend on the number and type of sequences, distance between them and the proteins and complexes surrounding that region. Furthermore, each of the four mammalian paralogs comprised within the CPEB family (herein CPEB1-4) have also been attributed different degrees of affinity with the above mentioned sequences (Fernández-Miranda and Méndez, 2012). This is due to the fact that they all share common domains, such as the NTD (N-terminal regulatory domain) and CTD (RNA-binding C-terminal domain) ones. While CTD remains largely conserved across the family, NTD is highly variable regarding its RRM (RNA-recognition motifs), and strongly susceptible to several post-transcriptional modifications (Mendez et al., 2000; Theis et al., 2003; Drisaldi et al., 2015), which leads to contemplate the fact that the different CPEBs will trigger different signals over a same mRNA target, thus engaging distinct expression patterns. To add up another level of complexity, it has been described by Igea and Mendez (Igea and Méndez, 2010; Hu et al., 2014) that some CPEBs (particularly CPEB1 and CPEB4) are able to self-regulate their expression using the CPE elements sheltered in their very own mRNA. This set of facts, adding up to the empirical behavioral observation of the different CPEBs in different tissues and disease stages, suggests that this family of proteins is capable to accurately adjust spatial and temporal signals in such a complex and precise manner; there is no doubt that there’s a long way ahead until we can start elucidating the specific mechanisms and circumstances under which CPEBs operate.

Early studies on the CPEB family members’ sequences show close similarities between CPEB2-4 compared to CPEB1, which have been clustered into a separated subfamily (Fernández-Miranda and Méndez, 2012; Ivshina et al., 2014). Intriguingly, CPEB orthologs have been found to be better conserved across different species than between paralogs (Wang and Cooper, 2010), for what we can assume their identities had a strong role to be selectively maintained through evolution, although the number of CPEBs within their family can vary between species. The first investigations on CPEBs used *Xenopus laevis* oocytes as a model to study cytoplasmic polyadenylation dependent translation. Later on, CPEB investigations began to use other models and move across other fields, unraveling different identities and roles for each CPEB member, all of them regarding the regulation of the poly(A) tail. CPEB1 has been the most studied member of this puzzling family, partly because it was the first to be discovered. Studies from Mendez lab have shown that CPEB1 can act both as an activator and a repressor of mRNA translation, depending on its phosphorylation status (Fernández-Miranda and Méndez, 2012); while the activator mechanism has been quite well characterized, the repression mechanism (when CPEB1 is unphosphorylated) remains debatable. CPEB1 will change its affinity for F (cleavage polyadenylation specific factor) upon phosphorylation, following PARN (poly(A) ribonuclease) eviction from the complex, and enabling Gld2 (a poly(A) polymerase) to enter the scene and begin polyadenylation during the meiotic (Kim and Richter, 2007) and mitotic stages (Novoa et al., 2010). CPEB1 has also been found to be implicated in cellular senescence (Burns and Richter, 2008), tumor development (Nagaoka et al., 2016), inflammation (Ivshina et al., 2015), synaptic plasticity (Udagawa et al., 2012), and liver homeostasis (Alexandrov et al., 2012), beyond meiosis. Conversely, CPEB2 was suggested some years ago to act as a translational repressor upon the elongation phase, via interaction with eEF2 (Chen and Huang, 2012), and very little information has been obtained on possible activation mechanisms on specific target mRNAs (Hägele et al., 2009). We know CPEB2 is expressed in the liver, as well as in the brain and in testis, that it interacts with b-catenin and CaMKII (which are both targets of CPEB1) (Turimella et al., 2015), and that it could be involved in HIF1a activity regulation, at least in neuroblastoma cells (Hägele et al., 2009). To date, the knowledge on CPEB2 and its role in cancer and liver is still modest but not less deserving of further research. CPEB3, in turn, has been mainly studied in the frame of synapse activity, where studies indicate this member of the family can both enhance activation and degradation of target mRNAs upon either monoubiquitinylation (Pavlopoulos et al., 2011), either cleavage by Calpain2 or either by forming prion-like aggregates (Drisaldi et al., 2015; Fioriti et al., 2015; Stephan et al., 2015). CPEB3 has been related to tumorigenesis and it seems it would also play a role in HCC progression (Zhang et al., 2020), but again, very little has been researched on that matter regarding CPEB3.

Ultimately, CPEB4 was also attributed repression and activation roles regarding cytoplasmic polyadenylation in specific contexts, such as terminal erythroid differentiation (Hu et al., 2014), circadian rhythms (also involving CPEB2) (Kojima et al., 2012; Maillo et al., 2017), oocyte maturation, somatic cell cycle and tumor progression and malignancy (Novoa et al., 2010; Ortiz-Zapater et al., 2012), cell survival (Kan et al., 2010; Chang and Huang, 2014), and pathological angiogenesis in the liver context (Calderone et al., 2016), strongly indicating a pro-tumoral role in cancer progression (Han et al., 2015; Hu et al., 2015; Zhong et al., 2015; Boustani et al., 2016), despite some reports pointing toward opposite directions (Peng et al., 2014).

## Cytoplasmic Polyadenylation Element Binding Proteins in Liver Disease

The liver is a complex organ, just as the family of proteins we’re writing about. It is also the most affected organ by the aging population and the modern lifestyle in industrialized societies, often fueling chronic liver diseases, which are currently still an underestimated and growing global public health problem. The epidemiology of liver disease is diverse; alcohol abuse, HCV/HBC viral infections and non-alcoholic fatty liver disease (NAFLD) are the most common causes, and studies cannot find agreement in which of them is the most prevalent one. One must note, before keeping on with this review, that the trend in the clinical field for the past years has strived to redefine NAFLD so that this affection can be rather diagnosed by inclusion criteria instead of exclusion criteria; this is how “metabolism-associated fatty liver disease” (MAFLD) is born, a term recently coined by an international panel, with growing popularity in literature (Eslam et al., 2020; Yamamura et al., 2020). This set of chronic liver diseases affects no less than 5.5 million people worldwide (Pimpin et al., 2018), with NA/MAFLD leading the rank, estimated to affect approximately 25% of the world population (Younossi et al., 2019). This affection, while remaining untreated, will continue to progress to non-alcoholic steatohepatitis (NASH), which very often will derive in serious liver injury stage by stage until reaching cirrhosis or even HCC. Associated metabolic risk factors will range from hypertension and dyslipidaemia, obesity and diabetes (Neuschwander-Tetri, 2010); cardiovascular disease is the most common cause of mortality in individuals affected by NA/MAFLD, followed by HCC (Adams and Angulo, 2005). However, when the patient reaches the stage of cirrhosis, liver disease will prevail as the number-one risk for mortality.

**FIGURE 2:**
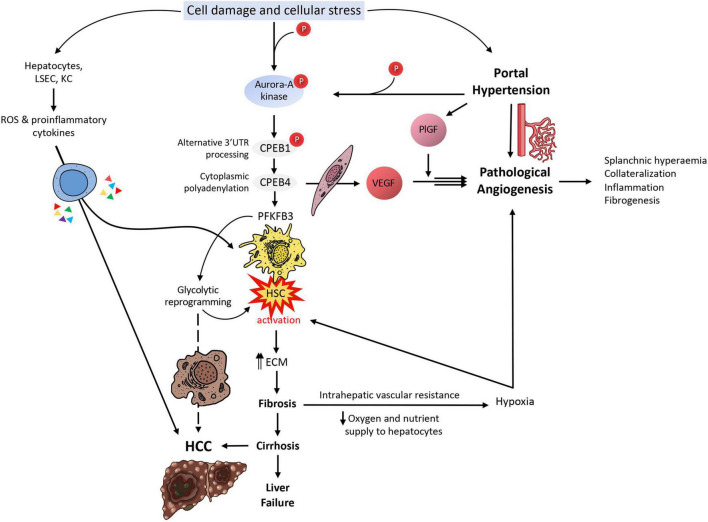
CPEB-mediated translational control in liver disease. Upon cell damage and cellular stress in the liver, Aurora-A kinase is phosphorylated; Aurora-A will in turn phosphorylate CPEB1, which will process mRNAs in their alternative 3′UTR during a first wave of translational activation. One of these mRNAs is CPEB4 mRNA; in a second wave of translational activation, CPEB4 will enhance the polyadenylation of several mRNAs, such as VEGF and PFKFB3, besides its own mRNA. VEGF will cause pathological angiogenesis and its effect will be greatly boosted by PlGF, leading to splanchnic hyperemia, collateralization of blood vessels, inflammation and fibrogenesis. These will cause an increase in portal hypertension, which will increase PlGF levels and increase Aurora-A kinase phosphorylation, both feeding back the VEGF loop. PFKFB3, on the other side, will cause the activation of HSCs, which will start synthesizing ECM elements that will contribute to liver fibrosis and later cirrhosis, if unresolved. Liver fibrosis will cause intrahepatic vascular resistance and thus a reduction in oxygen and nutrient supply to hepatocytes, causing them to enter a hypoxic state; upon this situation, hepatocytes will produce angiogenic factors, attempting to resolve the situation, but actually making it worse. On the other hand, cirrhosis is well-known for being the most common prelude for HCC. The glycolytic switch caused by the overexpression of PFKFB3 in this specific context might likely facilitate the survival of transformed cells in a hypoxic millieu. Hepatocytes, LSECs and KCs will also respond to cell damage and cellular stress by producing ROS and liberating proinflammatory cytokines, contributing to the activation of HSCs and the progression of HCC.

In all of these phases of the chronic liver disease, we find a cytoplasmic polyadenylation binding protein (CPEB) involved, acting in one way or another (Figures 1C, 2). This review aims to put together the investigations from the last decades on this family of proteins to shed some light on the purpose of their role in this particular organ, and to show how significant research is on this field, given the current inevitable background of these diseases, the magnitude of people affected by them, and the socioeconomic and health burden they imply. But before tackling the multiple roles the CPEB proteins play in chronic liver diseases, it might be useful to first have a brief insight into the cell composition, structure and basic physiological functions of this organ, to have a better global understanding.

The liver is the most extensive organ in our body after the skin, weighting around 2–3% of the whole bodyweight. Its function is essential for the body’s homeostasis, participating in the most important biological mechanisms amongst which stand detoxification of waste compounds, erythrocyte recycling, synthesis and secretion of bile, plasma protein synthesis and energetic metabolism homeostasis, among others (Fernandez, 2015). Because of its detoxification function, the liver is constantly being exposed to toxins and stress-inducing molecules, which greatly favor the staging of chronic liver disease, especially under an excess calorie intake and nutritionally imbalanced diet (mostly in the form of carbohydrates and fat) accompanied by a sedentary lifestyle. The most abundant cell population in the liver are hepatocytes (around 80%), their distribution shaping polygonal conformations organized in radial layers; the space between these layers is known as the liver sinusoid, where the nutrient exchange takes place. The rest of cells (20%) are grouped into the so-called non-parenchymal type: LSEC (liver sinusoidal endothelial cells), HSC (hepatic stellate cells), KC (Kupffer cells). LSECs are highly specialized cells which are found enclosing the liver sinusoid. They are characterized by being highly permeable and forming fenestrations in order to facilitate the process of nutrient delivery to hepatocytes. In addition, they are in charge of maintaining a low portal pressure regardless of global pressure fluctuations, and also maintain HSC quiescent to repress fibrogenesis. HSCs are pericyte-like cells found within the space of Disse (subendothelial area contained between LSEC and hepatocytes, where the exchange of molecules is given); their main function is stocking up on vitamin A, but they also play a big role in immune response over stress, besides mediating injury response and tissue regeneration. Finally, KCs are resident macrophages whose function is to process aged erythrocytes and other circulating waste, besides coping with immunological imbalance in the liver, when in need. Structurally, the liver tissue is mostly composed of multiple parcels named portal triads, which comprise branches from the hepatic artery and portal vein, and a bile duct; understanding this architecture is essential to follow the stages of disease development.

### Cytoplasmic Polyadenylation Element Binding Proteins and Cellular Stress

After a life of poor diet habits and physical inactivity, the liver starts to be forced to overcompensate for the cellular damage and dysfunction derived from oxidative stress over a net accumulation of energy in the form of triglycerides. Hepatocyte injury and death are at the center of the progression of NA/MAFLD to NASH, since they amplify inflammatory and fibrotic signaling in the pericellular milieu (Ibrahim et al., 2018). It is still unclear how the vicious cycle of progressive destruction and regeneration starts and the role that CPEBs have in it, but a study from Maillo et al. (2017) could be useful to bring some light over how CPEBs are involved in the pathogenesis of NA/MAFLD. Their work (Maillo et al., 2017) focuses on CPEB4, whose mRNA levels are intriguingly regulated in a circadian manner in the liver. This is also the case of CPEB2 (Kojima et al., 2012), although this study leaves it unrelated to hepatosteatosis. The absence of CPEB4 in high fat diet (HFD)-fed mice, though, resulted in exacerbated steatosis, sometimes accompanied by fibrosis, originated by lipid accumulation in the liver and impaired lipid metabolism.

The liver is complexly governed by a cell-autonomous circadian clock, which regulates the unfolded protein response (UPR) in hepatocytes. The maintenance of endoplasmic reticulum (ER) integrity by the UPR is crucial for glucose and lipid metabolism homeostasis; thus, alterations in UPR pathway are known to lead to hepatosteatosis and possibly type-2 diabetes. Although CPEB4 protein levels are not circadian themselves, its mRNA levels oscillate, generating a circadian mediator of UPR in order to anticipate periods of elevated ER overexertion (Maillo et al., 2017). CPEB4 and UPR are closely related because in hepatocytes under metabolic stress, such as the one induced by HFD, the ER triggers the UPR to maintain tissue homeostasis; eIF2a is then phosphorylated and global protein synthesis attenuation follows, in order to help the cell to adapt to the ER stress. However, p-eIF2a will selectively increase the translation of upstream open reading frame (uORF)-rich transcripts, amongst which stands CPEB4 mRNA (which actually contains eight uORFs). In a second wave of translational activation, CPEB4 will therefore bind CPE-containing mRNAs, encoding for multiple chaperones and other proteins involved in ER homeostasis and stress resolution.

Under CPEB4 depletion in mice, it is argued that impaired mitochondrial fatty acid oxidation and respiration will cause lipid accumulation and toxicity, besides also favoring the induction of apoptotic UPR branch, failing to adaptively attenuating HFD-induced ER stress. In addition, HFD-fed and CPEB4 KO mice showed hyperglycemia, and although CPEB4 KO was not directly related to glucose metabolism, it is indirectly associated to it through impaired lipid homeostasis, as the accumulation of lipids has an inhibitory effect on hepatic insulin signaling (Maillo et al., 2017), leaving liver gluconeogenesis unaffected. The authors of the study point out that their results indicate a cell-autonomous defect in hepatocytes, rather than a metabolic impairment in adipose tissue regarding the pathological response to HFD. One can fathom that CPEB4 depletion will induce hepatosteatosis as a result of unfolded protein accumulation, according to this study.

Other interactions described in literature that may potentially play a role in chronic liver disease initiation are, for example, CPEB1 on PTEN and STAT3 mRNAs; though CPEB1 interacts in a direct manner with these CPE-containing mRNAs by repressing them, in the absence of CPEB1 these factors become upregulated, interfering in glucose metabolism and causing insulin resistance in the liver in response to stressful ongoing events, such as high fat diet (Alexandrov et al., 2012). In addition, this interaction has been associated to elevated IL-6 serum levels in the same study upon CPEB1 knock-out mice; this finding is known to be correlated to insulin resistance and even cancer (Fève and Bastard, 2009). This complex interplay between pro-inflammatory and insulin-related factors will, if not cause, trigger and fuel stressful metabolic conditions in hepatocytes that might be hard to overcome (Kucukoglu et al., 2021).

### Cytoplasmic Polyadenylation Element Binding Proteins and Portal Hypertension

Portal hypertension (PHT) is usually one of the most significant and devastating complications of chronic liver disease, often silently manifesting at early stages of the disease. The profound hemodynamic disturbances are a consequence of vascular architecture distortion, and they are not limited to intrahepatic circulation as one would think; it is a fact that they will eventually involve the splanchnic and systemic vascular beds, characterized by a pathological increase of blood flow and consequent portosystemic collateral vessel formation in the form of pathological angiogenesis (Fernandez et al., 2016). It is in this scenario of PHT and pathological angiogenesis where we find a CPEB enrolled again.

**FIGURE 3:**
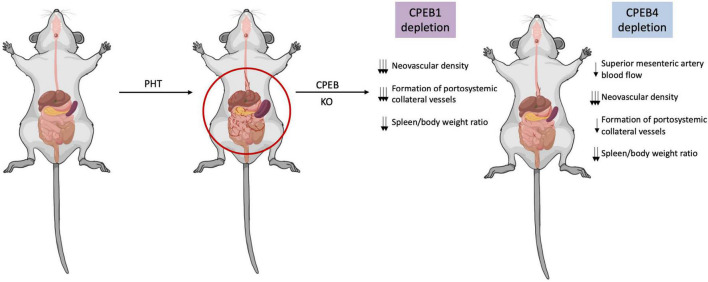
CPEB silencing limits pathologic angiogenesis in portal hypertension and protects against disease progression. In a context of portal hypertension (PHT), pathological neovascularization increases significantly contributing to increase splanchnic blood flow and portosystemic collateral vessels. Upon CPEB1 or CPEB4 depletion in animal models of PHT, splanchnic neovascular density is markedly and significantly reduced, while preexisting vasculature density remains untouched. The formation of portosystemic collateral vessels is also diminished after CPEB1 or CPEB4 depletion in a context of PHT. CPEB1 or CPEB4 ablation also prevents the PHT-induced increase in the spleen/body weight ratio, suggesting attenuation of portal hypertension.

Upon hemodynamic shear and mechanical stress trigger, increased blood flow and vascular growth factors, there is a rapid phosphorylation of Aurora-A kinase, a serine/threonine kinase that is able to activate CPEB1. Activated CPEB1 will then initiate alternative nuclear processing within 3′UTR of vascular endothelial growth factor (VEGF) and CPEB4 mRNAs; the latter activating cytoplasmic polyadenylation of VEGF mRNA, which will rapidly rise its translational rate (Figures 1C, 2). VEGF will activate endothelial cells to turn on mobility and filopodia protrusion in order to form tip cells and to initiate new sprouts (Fernandez et al., 2016). These cells are supported by stalk cells, which are also activated endothelial cells whose function is to establish the vessel’s lumen. Endothelial cells will then begin to secrete platelet-derived growth factor (PDGF) to attract pericytes and smooth muscle cells to stabilize the nascent vessel. This elaborated mechanism contributes to increase splanchnic neovascularization and splanchnic blood flow during PHT and chronic liver disease. It also participates in the formation of portosystemic collateral vessels, which try to alleviate the increased portal pressure by redirecting the enhanced portal venous inflow through the new collaterals. This angiogenic mechanism involving CPEB1 and CPEB4 overexpression is purely pathologic and plays a big role in quite every stage of the chronic liver disease; in cirrhotic patients, for instance, angiogenesis will contribute to the establishment and maintenance of abnormal hepatic architecture, perpetuating PHT and promoting fibrogenesis and inflammation. In this sense, CPEB silencing ameliorates major hallmarks of PHT, such as portosystemic collateral vessel formation, mesenteric arterial hyperdynamic circulation, and several surrogate markers of disease severity, such as increased von Willebrand factor plasma levels and splenic enlargement and hyperactivation, according to animal models (Calderone et al., 2016; Figure 3).

### Cytoplasmic Polyadenylation Element Binding Proteins and Neovascularization

For many years, research has focused on the overproduction of VEGF as the target for new therapies, but the problem with these drugs is that they target both physiological and pathological angiogenesis, and their use becomes restricted because of significant side effects, such as collapsing normal vasculature, leakage and bleeding (Calderone et al., 2016). In this sense, the research on the posttranscriptional mechanisms mediated by CPEBs opens a new door to target pathological angiogenesis solely.

Pathological angiogenesis is a process where new vessels sprout and branch from preexisting blood vessels (Ramirez-Pedraza and Fernández, 2019), and becomes clinically relevant in a context of PHT and cirrhosis, where CPEB1 and CPEB4 are upregulated in a similar way than the previously mentioned. Even though VEGF is directly guilty for launching neovascularization processes, it does not discern between a physiological or a pathological setting; but CPEBs do. Analysis on VEGF’s mRNA uncovered multiple regulating elements with diverse functions; the already known CPE domains at the 3′UTR allow CPEB1 and CPEB4 binding, and so do the PAS (putative polyadenylation site) domains. In addition, important feed-back loops where CPEB4 binds its own CPE boosts its translation and will surely cause an additional increase on VEGF overexpression. Otherwise, other AU-rich elements (AREs) will act as negative regulators of VEGF mRNA when its synthesis is not needed (Calderone et al., 2016).

When VEGF is overexpressed by hepatocytes, it is released into the extracellular matrix with the aim of recruiting endothelial cells and promoting their proliferation and differentiation to start creating new vessels, whereas when VEGF is produced by endothelial cells, it will alternatively strengthen their angiogenic phenotype. Knock-down of either CPEB1 or CPEB4 in endothelioma cells causes a reduction in VEGF protein expression, leaving its mRNA levels untouched (Calderone et al., 2016). This is translated in a halt of tubular-like structure formation *in vitro*. In knock-out mice, physiological angiogenesis will remain unharmed and global vasculature unaffected by CPEB depletion. In addition, induction of CPEB1 knock-out compared to induced CPEB4 knock-out reveals different preferences for different PAS domains, leading to a longer/shorter 3′UTR of CPEB4 and VEGF mRNAs. In the absence of the nuclear CPEB1 activated form, PAS2 will be the default polyadenylation site for CPEB4 and VEGF mRNAs; this domain will originate the longest 3′UTR transcript variant, containing more AREs and microRNA-binding sites, which will repress CPEB4 cytoplasmic translation, in this case. Removing these motifs from the CPEB4 transcript will cause CPEB4 mRNA stabilization and activation.

Vasculogenesis is in turn another major hallmark of chronic PHT that contributes to disease aggravation, modestly differing from the pathogenic angiogenesis above described (which is initiated by mature endothelial cells that activate upon PHT and start to proliferate). Vasculogenesis is originated by vascular stem/progenitor cells (VSPC), harbored in healthy mesenteric vessels before PHT steps in. Upon an unresolved increase in blood pressure, these cells will start to proliferate and differentiate into different vasculogenic types (such as mature endothelial cells – ECs – or – smooth muscle cells – SMCs – lineages) in order to trigger the formation of abnormal vessels (Garcia-Pras et al., 2017). CPEB4 is a considerable factor regarding the proliferation and proper differentiation of VSPC; Garcia-Pras and colleagues argue that the underlying molecular mechanism is probably mediated by two sequential and non-redundant translational waves again implicating CPEB1 and CPEB4, resulting in the upregulation of hundreds of mRNAs encoding factors that are differentially expressed during cell cycle. *In vivo* evidence with knock-out mice is comparable to the prior mentioned study (Calderone et al., 2016) and essentially agrees on the same terms. Garcia-Pras and colleagues observed that mesenteric CPEB4 levels correlate spatiotemporally with VSPC expansion and neovascularization under PHT circumstances, and *in vitro* studies determined that CPEB4 is critical for cell division, SPC function and pathological angiogenesis in cancer and PHT. In addition, the differentiation potential of VSPCs is subordinated to multiple factors including VEGF and PDGF and their corresponding receptors, which are in turn also upregulated in a PHT environment (Garcia-Pras et al., 2017).

### Cytoplasmic Polyadenylation Element Binding Proteins and Fibrosis

Unresolved and sustained insults in the liver with settled PHT will often entail scarring processes and consequent dysregulation of extracellular matrix (ECM), tipping the scale in favor of excessive matrix deposition. In addition, the nascent hypoxic areas due to distorted liver structure will also enhance angiogenesis and neovascularization attempting to resolve the ongoing condition, and far from making it better, it will strongly worsen the already established feedback loop of PHT-angiogenesis-hypoxia-fibrosis, rapidly and irreversibly deteriorating the patient’s health.

Fibrosis is described by Mejias et al. (2020a) as the abnormal and excessive deposition of collagen-rich ECM, resulting in compromised tissue and organ structure, and it is the sole histologic feature of NASH that currently anticipates clinical outcomes. In this advanced stage, angiogenesis is now stimulated by hypoxia, which is in turn caused by overcompensated tissue repairing processes in response to continuous insults to the liver. Wound healing responses will also trigger inflammation processes that will attract macrophages and activate HSC upon secretion of proinflammatory cytokines.

At a molecular level, the phosphorylation of Aurora-A kinase in the liver territory within a PHT context keeps activating translational waves implicating CPEB1 first and CPEB4 later. We saw how intimately related were VEGF and CPEB4 in pathological angiogenesis and neovascularization; the same happens in fibrogenesis, where CPEB4 binds the CPE sequences in PFKFB3′s mRNA, upregulating its translation and driving HSC activation and the expression of fibrogenic markers (Mejias et al., 2020b). In addition to this molecular mechanism, other cell populations (hepatocytes, LSECs, and KCs) will generate ROS and proinflammatory cytokines under cellular stress, also contributing to HSC transdifferentiation. Activated HSC shift into a myofibroblastic phenotype, characterized by being highly proliferative and producing and secreting ECM; VEGF-A and MMP9 are, among others, two factors that will mediate fibrosis-associated angiogenesis within the early stages. This highly proliferative HSC phenotype demands lots of energy to maintain its function, and it is fueled by PFKFB3-mediated glycolytic reprogramming as an additional energetic and synthetic supply. This metabolic shift frequently anticipates an advantage in many malignant transformed cells; following this trend, it is not crazy to assume that this mechanism will be later utilized by transformed HCC cells, in a scenario where the cirrhotic liver succeeds at evading global liver failure for a while.

Mejias and colleagues analyzed CPEB4 and PFKFB3 trends in human and mouse primary HSC cell lines and livers from cirrhotic patients and rat and mice experimental models, which showed correlating overexpressed levels upon HSC activation. Following this observation, they tested different approaches to understand the transdifferentiation mechanism and the main characters playing in it. From this study (Mejias et al., 2020b), they confirmed that (a) PFKFB3 antagonists inhibit dependent glycolysis and CPEB4 overexpression is attenuated in HSC, for what PFKFB3 drives glycolytic switch in HSC, (b) CPEB4 knock-out prevents PFKFB3 overexpression (and thus HSC activation) for what PFKFB3 is a direct target of CPEB4, and (c) CPEB4 silencing in HSC during liver injury fails at increasing PFKFB3 levels for what translational regulation by CPEB4 outranks transcriptional control (Figures 1C, 2).

Of course, and given this intricate context, the amount of activated signaling pathways and markers becomes huge and complex, involving multiple cell types, simultaneously cross-talking in different directions and chaotically influencing each other, which makes it difficult to establish a clear sequential script on the progression of liver disease. But at the same time, it represents an advantage in this specific context; targeting a single moiety, CPEB4 in this case, would synchronously act on all the processes involved in the progression of chronic liver disease.

### Cytoplasmic Polyadenylation Element Binding Proteins and Hepatocellular Carcinoma

Hepatocellular carcinoma (HCC) will be the last stage in chronic liver disease, commonly appearing in cirrhotic patients before fatal liver failure. In addition, a significant share of NASH-related HCC thrives in livers unaffected (or minimally affected) by fibrosis (Kucukoglu et al., 2021). Together, it is globally estimated that HCC is the first primary liver cancer and the third most usual cause of cancer-related mortality (Chalasani et al., 2018). We have seen a correlation between chronic inflammation caused by a poor diet, but mechanisms linking NA/MAFLD and NASH to HCC are still poorly understood. At a molecular level, this is most likely initiated by unresolved ROS and ER stress, which can fuel tumor cell proliferation through UPR pathway (Jialal et al., 2014; Engin, 2017) as previously explained. In a fat-rich environment, increased adipokines and proinflammatory cytokines will often perpetuate chronic inflammation via TNFa, IL-6 and activation of NF-kB, which contribute to inhibit apoptosis in the liver and promote proliferation, invasion and metastasis (Lin et al., 2010; Kucukoglu et al., 2021).

As translational mediators, CPEBs are involved in many processes related to the cell cycle progression, such as cellular division, differentiation and senescence, among others. The only CPEB so far expendable to the mitotic cell division is CPEB3 (Giangarrà et al., 2015). In a global cancer context, aberrant CPEB expression has been linked to cell proliferation, invasion, malignant transformation and angiogenesis through translational reprogramming in numerous types of cancer (D’Ambrogio et al., 2013), indicating that mRNA processing is important for tumor growth; however, not all CPEBs play the same role in tumorigenesis. According to literature, CPEB1 and CPEB3 act as tumor suppressors, while CPEB2 rather displays oncogenic features (Chen et al., 2016); in contrast, CPEB4 remains to be classified into one of these two categories. Thus, ectopic or imbalanced levels of CPEB subtypes may modulate the behavior of cancer cells and tilt the cell fate toward tumor development instead of senescence or controlled proliferation (Fernández-Miranda and Méndez, 2012; Ortiz-Zapater et al., 2012; Giangarrà et al., 2015).

Despite the diverse roles CPEBs play in different cancer types, very limited knowledge has been gathered regarding HCC. While CPEB4 is physiologically expressed in the brain, heart, kidney and lung (Ortiz-Zapater et al., 2012), it has been the most related CPEB to HCC, although its role comes as quite paradoxical; whilst it seems to play a tumor-suppressor role in HCC, it’s been described as an oncogenic promoter in other kinds of cancer (Tsai et al., 2016). Observations of CPEB4 levels in different HCC stages suggest CPEB4 could play a phase-dependent role in HCC; but whether CPEB4 can be considered as a diagnostic marker or therapeutic target in HCC needs to be further researched. *In vitro* studies on liver cancer cells such as HepG2 revealed that CPEB4 knock-out promotes colony formation, and CPEB4 knock-down accelerates growth in xenograft mice (Tsai et al., 2016). Tsai and colleagues also collected and analyzed data from 49 human HCC samples, where they found CPEB4 to be mostly overexpressed in early stages of HCC and greatly decreased in late stages, suggesting CPEB4’s role at later stages of HCC intends to facilitate HCC progression, manifesting a complicated biphasic role in tumorigenesis. Other molecular mechanisms implicating CPEB2 and mostly CPEB1 have been described for example by Nairismagi and colleagues (Nairismägi et al., 2012), regarding the role of Twist1 in epithelial-to-mesenchymal transition (Yang et al., 2004). This transcription factor seems to be repressed by CPEB1 and CPEB2 in physiological conditions, and upon lack of these CPEBs, it is then up-regulated, promoting E-cadherin loss and inducing cell migration. Although this mechanism has not yet been studied in a HCC context, it is worthy to bear in mind that Twist1 is activated under hypoxic circumstances (Sun et al., 2009), when HIF1a is also active and not repressed by CPEB1 and CPEB2 (Hägele et al., 2009; Chen and Huang, 2012), a situation that could be perfectly given in a tumoral and fibrotic environment lacking nutrient and oxygen influx. Another manner CPEBs influence HCC progression is through miRNAs, a booming field in the past decade. Some studies have been able to find multiple miRNA binding sites in CPEB mRNAs (Morgan et al., 2010) suggesting a regulating role of CPEB functions (Richter, 2007). A quite recent study by Zou and colleagues (Zou et al., 2016) describes a mechanism of cell proliferation, migration and invasion enhancement in HCC, concerning CPEB3 and miRNA-107. According to this study, miRNA-107, which is implicated in various cancers (Song et al., 2014; Zhou et al., 2014; Tao et al., 2015), was overexpressed in Huh7 and HepG2 cell lines, hindering CPEB3 mRNA and protein levels through binding to its 3′UTR. This CPEB3 down-regulation was accompanied by an increase in p-Akt and EGFR levels and a decrease in p21 levels, assigning a tumor-suppressor role for CPEB3 in a context of HCC.

### Cytoplasmic Polyadenylation Element Binding Proteins and the Immune System

Non-alcoholic or metabolic associated fatty liver disease is very often attributed an immune pro-inflammatory component as one of the underlying mechanisms involved in disease progression, but to which extent is it? And more importantly, are CPEBs again involved in immune dysregulation in chronic liver disease? There are solid evidences of altered immune cell behavior in different stages of chronic liver disease, and a couple of studies have found a link to CPEBs for now.

In 2015, Richter’s lab was focusing on CPEB1’s role in inflammation (Ivshina et al., 2015), which was later confirmed and extended by Cui et al. (2020). In this study, they uncovered a mechanism by which depletion of CPEB1 causes a substantial increase in macrophage IL-6 synthesis, a well characterized pro-inflammatory cytokine with an active role both in chronic inflammation in a context of obesity and HCC progression (Alexandrov et al., 2012; Taniguchi and Karin, 2014; Ramirez-Pedraza and Fernández, 2019). In normal conditions, CPEB1 is bound to the CPE elements in TAK-1 mRNA, repressing the translation of the transcript. Upon CPEB1 depletion, TAK-1 is thus translated, causing the phosphorylation of IkBa, which will then dissociate from cytoplasmic NF-kB, allowing its internalization in the nucleus. NF-kB is a transcription factor that will activate the transcription of IL-6 in macrophages and polarized monocytes. The *in vivo* part of this study included HFD-fed mice; they observed that not only KO mice exhibited more IL-6 compared to WT, but also that HFD-fed KO mice presented insulin resistance, which has been many times related to chronic inflammation in literature, and also related to CPEB1 and CPEB2 depletion (Alexandrov et al., 2012). In the liver, KCs are the main producers of IL-6, and hepatocytes display high amounts of IL-6R, the IL-6 receptor; this causes them to be more susceptible to the up-regulation of signaling pathways such as JAK/STAT, MAPK/ERK and PI3K-Akt, all mediated and fueled by IL-6 (Taniguchi and Karin, 2014).

Conversely, our lab has been working for the past years in studying the role of CPEB4 in high fat diet induced obesity (Pell et al., 2021), and the results have elucidated another mechanism by which macrophages are altered by the adipose tissue, not previously described in literature. Indeed, CPEB4 drives a posttranscriptional reprogramming in adipocytes of white adipose tissue under obesity conditions. This rewiring stimulates the production and release of proinflammatory factors from obese adipocytes, which in turn promote a proinflammatory switch in macrophages and increase their migratory capacity. RIP-seq analysis from this study also revealed that CPEB4 is necessary for CCL2 and TLR4 production, which are implicated in the activation and recruitment of proinflammatory macrophages (Ramirez-Pedraza and Fernández, 2019) and also in liver fibrosis (Loomba et al., 2021). Moreover, the depletion of CPEB4 significantly attenuates CCL2 and TLR4 levels in adipose tissue, besides releasing higher levels of IL-10, an anti-inflammatory cytokine.

Activated pro-inflammatory macrophages are also related to pathological angiogenesis; it’s been described by Ramirez-Pedraza and Fernandez (Ramirez-Pedraza and Fernández, 2019) that they actively support the formation of new vasculature through the secretion of pro-angiogenic factors (such as TGF-b, VEGF, PlGF, PDGF and matrix-remodeling proteases) and physical interaction with sprouting areas in the liver. While VEGF upregulation is directly linked to new vessel formation as described in prior sections, PlGF, for instance, which is also upregulated in this context of chronic inflammation (Li et al., 2017), will amplify VEGF activity by modifying the binding affinity of VEGF’s receptors (Carmeliet et al., 2001; Fischer et al., 2007; Van Steenkiste et al., 2009). The nascent vascular network therefore allows macrophages to disseminate more easily throughout the tissue and interact with different cell types, guided by factors released by hepatocytes and often helping at HSC activation, promoting ECM accumulation and thus exacerbating fibrogenesis.

Other immune cells have been associated to chronic inflammation and many studies have described the cross-talk between the adipose tissue and the immune system (Gomes et al., 2016; Nati et al., 2016; Loomba et al., 2021); although macrophages have been at the spotlight of CPEB mediation of inflammatory processes regarding chronic liver disease and obesity until now, the door is open for other cell types, such as neutrophils and T-cells, to share an equally significant role in this scenario.

## Summary and Conclusion

Recent findings from our group and other researchers unveil previously unrecognized posttranscriptional regulatory circuits orchestrated by the RNA-binding proteins CPEBs, which are required for portal hypertension, neovascularization, steatosis and fibrogenesis in liver disease. This improved understanding may create new opportunities to develop better treatments to combat chronic liver disease. In this regard, a potential therapeutic approach would be to block the binding of the CPEB proteins to their target mRNAs. For that, in collaboration with other groups, we are currently developing small compounds with CPEB4 inhibitory activity. These compounds specifically block the RNA binding site for CPEB4, preventing the binding of CPEB4 to their target mRNAs. It will be important to deliver these small compound CPEB4 inhibitors to the specific cell type of interest. For that, we are also collaborating with experts to develop drug-carrier therapeutic strategies to specifically deliver small compounds to any cell of interest. It is hoped that these CPEB4 inhibitors will make it to the clinic in the near future.

## Author Contributions

AB wrote the manuscript and designed figures. MF wrote and corrected sections of the manuscript and got funding. Both authors contributed to the article and approved submitted version.

## Conflict of Interest

The authors declare that the research was conducted in the absence of any commercial or financial relationships that could be construed as a potential conflict of interest.

## Publisher’s Note

All claims expressed in this article are solely those of the authors and do not necessarily represent those of their affiliated organizations, or those of the publisher, the editors and the reviewers. Any product that may be evaluated in this article, or claim that may be made by its manufacturer, is not guaranteed or endorsed by the publisher.

## Abbreviations

A-site: acceptor site
Akt: RAC-alpha serine/threonine protein kinase, also known as PKB (protein kinase B)
ARE: AU-rich element
Calpain2: CaMKII, Ca^2+^/calmodulin-dependent protein kinase II
CCL2: monocyte chemoattractant protein 1
CDC2: gene encoding for Cyclin Dependent Kinase 1 (Cdk1), also called p34
CPE: cytoplasmic polyadenylation element
CPEB: cytoplasmic polyadenylation element binding protein
CPSF: cleavage polyadenylation specific factor (complex). It binds the AAUAAA/AUUAAA sequence and recruits PAP.
CTD: RNA-binding C-terminal domain
DAMPs: damage-associated molecular patterns
Dcp1: decapping enzyme 1
Dcp2: decapping enzyme 2
E-site: exit site
eEF1A-B: eukaryotic elongation factor 1A-B
eEF2α: eukaryotic elongation factor 2α
p-eEF2α: phosphorylated eukaryotic elongation factor 2α
EC: endothelial cells
ECM: extracellular matrix
eIF: eukaryotic initiation factor
eIF4E: eukaryotic initiation factor 4E
eIF4G: eukaryotic initiation factor 4G
eIF5-A: eukaryotic initiation factor 5-A
ER: endoplasmic reticulum
eRF1: eukaryotic release factor 1
eRF3: eukaryotic release factor 3
GLD-2: germ line development protein 2
GTP: guanosine triphosphate
HBV: hepatitis B virus
HCC: hepatocellular carcinoma
HCV: hepatitis C virus
HepG2: immortal hepatocellular carcinoma cell line
HFD: high fat diet
HIF1α: hypoxia-inducible factor α
HSC: hepatic stellate cells
Huh7: human hepatoma derived cell line
IκBα: NF-κB Inhibitor α, also known as major histocompatibility complex enhancer binding protein MAD3
IL-6: interleukin 6
IL-6R: interleukin 6 receptor
iKO: inducible knock-out
IRES: internal ribosome entry site
JAK/STAT: janus kinases/signal transducer and activator of translation pathway
KC: Kupffer Cells (macrophages)
KO: knock-out
LSEC: liver sinusoidal endothelial cells
m^7^Gppp: 5′cap
MAFLD: metabolic-associated fatty liver disease
MAPK/ERK: mitogen activated protein kinases/extracellular signal regulated kinases pathway
miRNA: micro RNA
MMP9: matrix metalloproteinase 9
NAFLD: non-alcoholic fatty liver disease
NASH: non-alcoholic steatohepatitis
NF-κB: nuclear factor κB p65 subunit
NTD: N-terminal regulatory domain
p21: protein 21
p34: protein 34
p-Akt: phosphorylated Akt
P-site: Peptidyl site
PABP: Poly(A) binding protein (family of proteins).
PAP: Poly(A) polymerase
PARN: Poly(A) ribonuclease
PAS: polyadenylation site
PDGF: platelet-derived growth factor
PFKFB3: 6-phosphofructo-2-kinase/fructose-2,6-biphosphatase 3
PHT: portal hypertension
PI3K-Akt: phosphatidylinositol-3-kinase-Akt pathway
PlGF: placental growth factor
PTEN: phosphatidylinositol 3,4,5-triphosphate 3-phosphatase and dual-specificity protein phosphatase
RBPs: RNA binding proteins
ROS: reactive oxygen species
RRM: RNA recognition motif
SMC: smooth muscle cells
STAT3: signal transducer and activator of transcription 3.
TAM: tumor associated macrophages
TAK1: TGF-β activated kinase 1
TGF-β: transforming growth factor β
TNFα: tumoral necrosis factor α
TLR4: toll-like receptor 4
Twist1: twist-related protein 1
uORF: upstream open reading frame
UPR: unfolded protein response
UTR: untranslated region
VEGF: vascular endothelial growth factor
VSPC: vascular stem/progenitor cells
WT: wild type
Xrn1p: 5′ to 3′ exoribonuclease 1 protein.
